# Substrate-dependent gene regulation of self-assembled human MSC spheroids on chitosan membranes

**DOI:** 10.1186/1471-2164-15-10

**Published:** 2014-01-05

**Authors:** Hsi-Yi Yeh, Bing-Hsien Liu, Martin Sieber, Shan-hui Hsu

**Affiliations:** Institute of Polymer Science and Engineering, National Taiwan University, and the Research Center for Developmental Biology and Regenerative Medicine, National Taiwan University, Taipei, Taiwan; Bionet Corporation, Taipei, Taiwan

**Keywords:** Mesenchymal stem cells (MSCs), Cellular spheroids, Chitosan, Calcium signaling, Gene profile, Microarray

## Abstract

**Background:**

Three-dimensional (3D) multicellular spheroids of mesenchymal stem cells (MSCs) are generally regarded to have beneficial properties over MSCs in monolayer. Recent literatures have documented that MSCs can self-assemble into 3D spheroids with a greater capacity for differentiation into various cell types when grown on chitosan (CS), a biopolymer. The genomic modulation occurring in these MSC spheroids is thus of essential importance for understanding their uniqueness and therapeutic potentials. In this study, 3D spheroids self-assembled from human umbilical cord MSCs grown on CS membranes were analyzed by mRNA as well as microRNA microarrays, which helped identify the critical signaling events that may alter the cellular functions during the spheroid forming process.

**Results:**

Genes screened from mRNA and microRNA cross-correlation analyses were further confirmed with the quantitative reverse transcriptase-polymerase chain reaction (qRT-PCR) analysis. Results revealed the regulation of a significant number of calcium-associated genes, which suggested the crucial role of calcium signaling in CS-derived MSC spheroids. In addition, many genes associated with the multilineage differentiation capacities and those associated with the antiinflammatory and antitumor properties of MSCs were upregulated. The genetic modulation was significantly more remarkable and endured longer for MSC spheroids derived on CS substrates compared to those derived on a non-adherent (polyvinyl alcohol) substrate.

**Conclusions:**

Based on the study, the culture substrates used to prepare 3D MSC spheroids may predefine their properties through cell-substrate interaction.

**Electronic supplementary material:**

The online version of this article (doi:10.1186/1471-2164-15-10) contains supplementary material, which is available to authorized users.

## Background

Mesenchymal stem cells (MSCs) are extensively used as the cell source for regenerative medicine because of their capacities to differentiate into different lineages and expand while maintaining their undifferentiated state. MSCs are commonly cultured as two-dimensional (2D) monolayer by traditional tissue culture techniques. However, the 2D culture method has difficulty in overcoming the replicative senescence and maintaining the self-renewal properties, which results in cells of low differentiation capacity [1]. A three-dimensional (3D) culture environment is considered more favorable than 2D monolayer culture for cell-cell contacts. Previous studies have developed several methods to generate 3D MSC spheroids. Many of these methods involve the use of cell suspension system or non-adherent surface to induce spheroid formation [2–4]. In general, these 3D MSC spheroids were reported to have greater differentiation capacities.

Chitosan (CS) is the deacetylated derivative of chitin which is abundant in shell of shrimp or crap, and in fungus, and the content is only next to cellulose in nature. CS has a main structure composed of β(1-4) linked D-glucosamine residues with different amounts of N-acetyl-glucosamine group. Owing to its biocompatibility and biodegradability, CS has been widely studied as a scaffolding material for tissue engineering. Recent findings have revealed that MSCs attached on the membranes made of CS can form self-assembled 3D cellular spheroids [5–7]. During the process, MSCs attach and spread on CS membranes before they retract their pseudopodia to form multicellular spheroids. This self-assembly process is quite different from that occurs in suspension or hanging drop systems, or on non-adherent polymer surfaces. Several genes/proteins have been referred to participate in the process of spheroid formation on CS, including cadherin molecules [8, 9], Rho/Rho-associated kinase (ROCK) [5], and the Wnt molecule [9]. Activations of these proteins were not as evident for spheroids on non-adherent surfaces. In addition, it was observed that the surface-bound calcium on CS substrates may be transported into MSCs and play a role in spheroid formation as well as gene regulation [9]. Although a few changes in gene/protein expression were observed, the exact mechanism for spheroid formation on CS is still far from being elucidated. Therefore, a more comprehensive understanding of the genomic profile for CS-derived MSC spheroids is essential for further revealing the substrate-dependent nature of these unique MSC spheroids.

The technique of microarray has been developed to detect the changes within cells and is a powerful tool by which many genes can be probed simultaneously. Dalby et al. have reported the genomic expression profile of human MSCs responding to the shape of their environment by the messenger RNA (mRNA) microarray [10]. The antiinflammatory properties of human MSC spheroids generated by hanging drop have also been compared to those of the adherent MSC monolayer by surveying with mRNA microarray [2]. Furthermore, recent advances in microRNA (miRNA), a class of non-coding small RNA, have identified a few important modulators in stem cell proliferation and differentiation. They can bind to the cognate mRNA to repress the expression of target genes. Simultaneous analyses of the mRNA and miRNA expression profiles may help narrowing down the signaling events involved in the behavior change of the cells [11].

In this study, we examined both mRNA and miRNA expression profiles of the CS substrate-induced 3D spheroids of human MSCs isolated from the umbilical cord, using 2D MSCs on tissue culture polystyrene (TCPS) as a control. Cross-correlation analysis of the results from these two microarrays was further confirmed with the quantitative reverse transcriptase-polymerase chain reaction (qRT-PCR) analysis to identify the critical signaling events for substrate-derived MSC spheroids during the spheroid forming process.

## Results

### Characteristics of human umbilical cord MSCs

The expression profile of cell surface markers analyzed by flow cytometry is shown in Additional file 1: Figure S1. Human umbilical cord MSCs were positive for specific antigen markers of MSCs such as CD13, CD29, CD44, CD59, CD61, CD71, CD73, CD90, CD105, CD166, and HLA-ABC, and negative for specific markers of endothelial cells and haematopoietic cells including CD14, CD34, CD45, CD133, and HLA-DR. Besides, cells showed positive expression of CD56 and low expression of CD106, which was consistent with that described for human umbilical cord MSCs in literatures [12, 13].

### Surface properties of CS membranes

**Figure 1:**
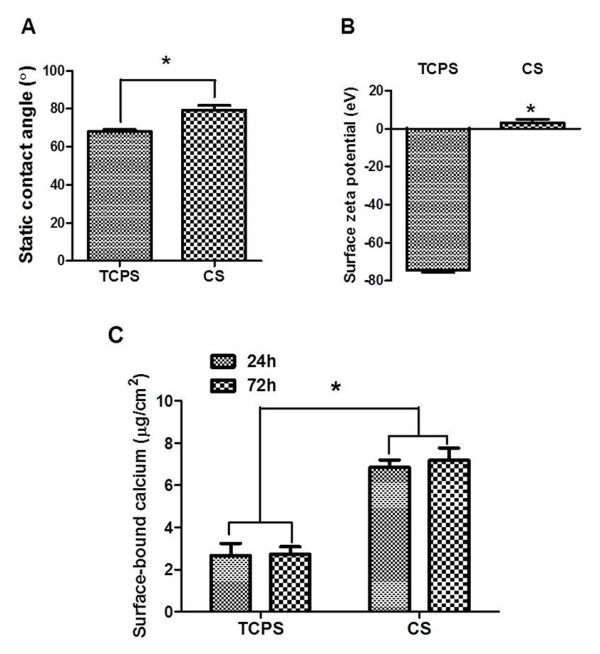
**Surface properties of cell culture substrates. (A)** The static contact angle of CS and TCPS. **(B)** The surface zeta potential of CS and TCPS. **(C)** The amount of surface-bound calcium on CS or TCPS after being soaked in the culture medium for 24 h without cells. * P < 0.05 among the indicated groups (n = 3).

**Figure 2:**
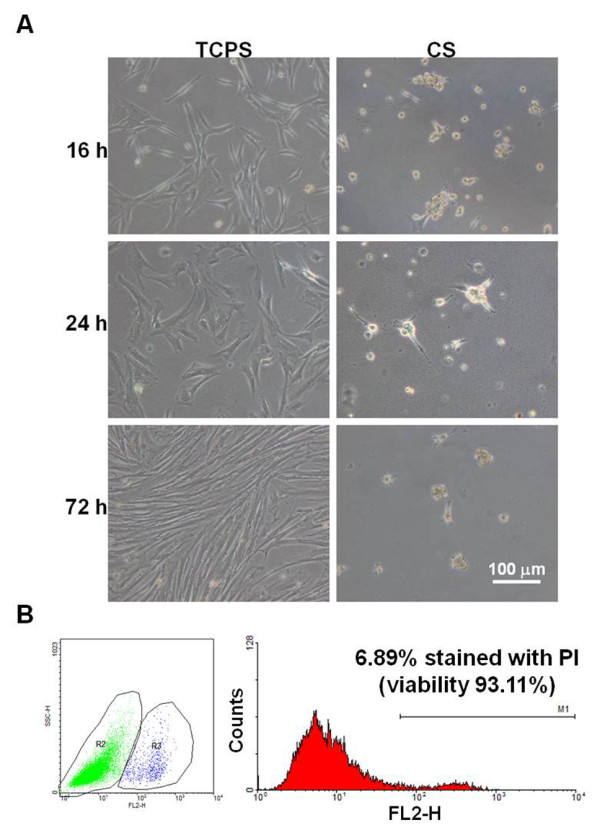
**Phenotype of MSCs grown on CS or TCPS. (A)** The morphologies of MSCs on CS or TCPS after 16 h, 24 h and 72 h. **(B)** The cell viability of MSC spheroids on CS after 24 h analyzed by flow cytometry. The percentage of cells without being stained by PI was defined as the cell viability.

The different surface properties of CS membranes and TCPS are summarized in Figure 1. The static water contact angle of CS membranes was 79.48±2.26° (n = 3) and that of TCPS was 68.02±1.13° (n = 3). The greater contact angle of CS vs. TCPS revealed the slightly higher hydrophobicity of CS, which may be attributed to the rearrangement of hydrophobic N-acetyl groups of CS to move to the surface and reduce the surface free energy while exposed to atmosphere. The surface zeta potential of CS was relatively neutral (3.16±1.74 mV, n = 3), distinct from the negatively charged TCPS (-74.34±1.22 mV, n = 3). Previous literature suggested an isoelectric point at pH 7.4 for CS arising from the deprotonation of positive amino group [14]. Another study indicated that the amino groups of CS could chelate calcium ion to form CS/calcium ion complexes (CS–NH_2_ · · · Ca^2+^) [15]. The amount of surface-bound calcium on CS membranes (Figure 2C) was significantly greater than that on TCPS after either 24 h or 72 h. The consistent values observed at 24 h and 72 h suggested a saturation of surface-bound calcium.

### Spheroid formation for MSCs growth on CS membranes

The morphology of MSCs grown on CS membranes and TCPS is shown in Figure 2. As was expected, MSCs attached to TCPS with fibroblast-like morphology. On the other hand, MSCs on CS remained attached to the surface before 16 h and formed spheroids afterwards. The average diameter of the MSC spheroids on CS membranes was 64.9 ± 9.5 μm at 24 h (n = 30). The spheroid size was relatively stable within 72 h (e.g. averaged 56.9 ± 14.3 μm at 72 h, n = 30). The cell viability for the MSC spheroids was 93.11% at 24 h.

### Cross-correlation analysis between mRNA and miRNA microarrays

**Figure 3:**
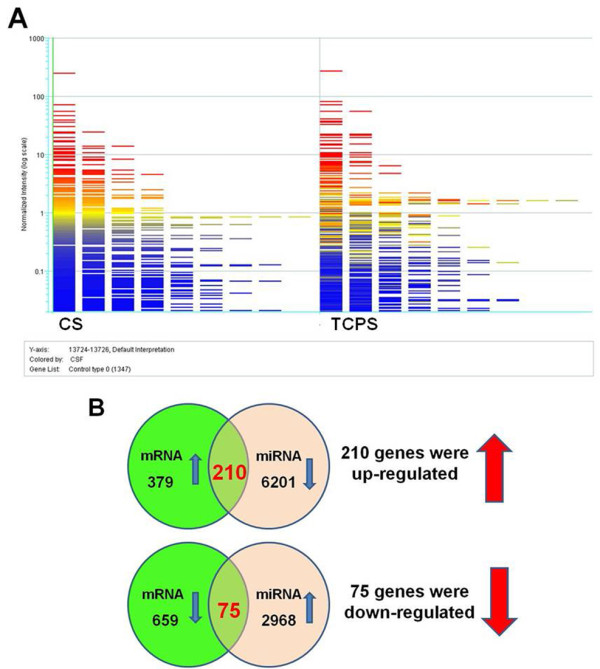
**The results of microarrays for MSCs grown on CS or TCPS. (A)** The screening of the miRNA microarray for MSCs grown on CS or TCPS. **(B)** The numbers of genes that were subjected to cross-correlation analysis of mRNA and miRNA microarrays and the numbers of screened genes based on the cross-correlation analysis. A complete list of screened genes as well as their expression ratios is shown in Additional file 2.

Based on the screening of the mRNA microarray, 589 upregulated genes and 734 downregulated genes showed significant difference (i.e. CS/TCPS ratio higher than two times or lower than a half, and the p-value lower than 1×10^-3^) for MSCs on CS vs. TCPS. On the other hand, screening based on the miRNA microarray showed that there were 6411 targeted genes corresponding to the downregulated miRNA, and 3043 targeted genes corresponding to the upregulated miRNA. Cross-correlation analysis of these two microarray data have helped narrowing down the critical genes involved in the behavioral changes of these cells. Screening based on the cross-correlation analysis with the software, Agilent.TwoColor.28004 (Agilent Technologies) revealed that there were 210 upregulated genes and 75 downregulated genes. The results of microarray analyses are shown in Figure 3. In addition, the screened genes as well as their expression ratio are listed in Additional file 2.

The biological significance behind the list of screened genes was further analyzed by the DAVID (the Database for Annotation, Visualization and Integrated Discovery) program to evaluate the gene enrichment in gene ontology (GO terms) and Kyoto Encyclopedia of Genes and Genomes (KEGG) pathway maps (Additional file 3). The results of gene enrichment in KEGG pathway maps suggested that several pathways were regulated for MSCs on CS vs. TCPS including calcium signaling pathway (hsa04020), focal adhesion (hsa04510), regulation of actin cytoskeleton (hsa04810), adherens junction (hsa04520), gap junction (hsa04540), ECM-receptor interaction (hsa04512), p53 signaling pathway (hsa04115), cytokine-cytokine receptor interaction (hsa04060), tryptophan metabolism (hsa00380), and TGF-β signaling pathway (hsa04350).

Among the 285 genes, we picked up the ones with high expression ratio, the critical ones in certain signaling pathways, or the ones which had been reported in literature to be associated with cell adhesion, migration, or fate decision. They were then further confirmed by qRT-PCR.

### Regulation of calcium-associated genes for MSCs on CS

A number of calcium-associated genes were noted and listed in Table 1 including those of calcium channels, receptors, and intracellular signaling proteins. ATP2B1 and ATP2B4 (plasma membrane calcium ATPase isoform 1 and 4) are highly regulated Ca^2+^ extrusion pumps and provide fine-tuning of intracellular calcium level [16]. SLC8A1 (often named as NCX1) is a Na^+^-Ca^2+^ exchanger which also modulates Ca^2+^ extrusion system from the cells [17]. TRPA1 and TRPC4 belong to the transient receptor potential (Trp) channel superfamily that regulates the mechanism for Ca^2+^ entry [18]. The upregulation of ATP2B1 and TRPA1 and downregulation of ATP2B4, SLC8A1, and TRPC4 for MSCs on CS vs. TCPS suggested that the process for intracellular calcium homeostasis was turned on. Modulation of these genes may cause an elevation in the intracellular calcium level.

**Table 1:** **The group of calcium signaling-associated genes screened from microarrays**

Gene symbol	Gene full name	Ratio (MA)	Ratio (qRT-PCR)
ATP2B1	ATPase, Ca++ transporting, plasma membrane 1	2.40	3.17 ± 0.88*
ATP2B4	ATPase, Ca++ transporting, plasma membrane 4	0.48	0.68 ± 0.23*
SLC8A1	solute carrier family 8 (sodium/calcium exchanger), member 1	0.22	0.40 ± 0.15*
TRPA1	transient receptor potential cation channel, subfamily A, member 1	4.46	24.58 ± 16.15*
TRPC4	transient receptor potential cation channel, subfamily C, member 4	0.21	0.02 ± 0.01*
HTR2A	5-hydroxytryptamine (serotonin) receptor 2A, G protein-coupled	10.36	26.92 ± 10.07*
HTR7	5-hydroxytryptamine (serotonin) receptor 7, adenylate cyclase-coupled	2.99	-
PDGFRA	platelet-derived growth factor receptor, alpha polypeptide	3.29	3.38 ± 1.17*
GPR68	G protein-coupled receptor 68	4.33	10.80 ± 1.42*
F2R	coagulation factor II (thrombin) receptor	2.26	6.98 ± 1.53*
BDKRB2	bradykinin receptor B2	2.59	-
EDNRA	endothelin receptor type A	2.27	-
ADRB2	adrenoceptor beta 2, surface	0.35	-
MAP3K8	mitogen-activated protein kinase kinase kinase 8	3.61	13.31 ± 2.04*
ITPR1	inositol 1,4,5-trisphosphate receptor, type 1	2.23	2.36 ± 0.32*
PLA2G4A	phospholipase A2, group IVA (cytosolic, calcium-dependent)	5.51	6.44 ± 2.70*
RASGRP3	RAS guanyl releasing protein 3 (calcium and DAG-regulated)	4.35	2.78 ± 0.90*
CALM2	calmodulin 2 (phosphorylase kinase, delta)	0.30	0.08 ± 0.05*

MA: based on microarray data.

qRT-PCR: based on quantitative RT-PCR analysis.

*P < 0.05 between CS and TCPS groups (n = 5).

A few genes of calcium-associated receptors were upregulated for MSCs growth on CS vs. TCPS. HTR2A and HTR7 (5-hydroxytryptamine receptor 2 A and 7) are the receptors for serotonin which is a well-characterized neurotransmitter with regulative function in multiple physiological aspects [19]. GPR68 (G protein-coupled receptor 68, also named as OGR1) is a proton-sensing receptor that can modulate the level of intracellular calcium [20]. PDGFRA (Platelet-derived growth factor receptor alpha) and F2R (thrombin-activated G protein-coupled receptor, and often named as the protease-activated receptor-1, PAR1) are also intracellular calcium modulators [21, 22]. The activation of these calcium-associated receptors may directly enhance the level of intracellular calcium and lead further to cytoskeleton rearrangement.

Several intracellular signaling genes were also upregulated. These genes included those of MAP3k8 (mitogen-activated protein kinase kinase kinase 8, also denoted tumor progression locus 2, Tpl2), ITPR1( inositol 1,4,5-trisphosphate receptor, type 1), RASGRP3 (calcium and diacylglycerol-regulated RAS guanyl releasing protein 3), PLA2G4A (cytosolic calcium-dependent phospholipase A2). MAP3K8 is required for the transduction of signals initiated by PAR1 and other G-coupled receptors, which promote actin reorganization and cell migration. MAP3K8 can also mediate signal-induced increases in cytoplasmic Ca^2+^ through the activation of phospholipase C [23]. On the other hand, ITPR1 is a Ca^2+^-release channel located on intracellular membranes, especially the endoplasmic reticulum (ER). The IP3 receptor has an affinity for IP3 in the low nanomolar range. Moreover, cytosolic Ca^2+^ is considered as a co-agonist of the IP3 receptor, as it strongly increases the IP3 receptor activity at concentrations up to about 300 nM [24]. RASGRP3 is a calcium and DAG-regulated RAS guanyl releasing protein which can activate small GTPases such as RAS and RAP1 [25]. PLA2G4A is a member of phospholipases A2 (PLA2s) superfamily, which regulates the release of arachidonic acid (AA) [26]. Although calmodulin is also a family of Ca^2+^ binding proteins and mediates many important cellular processes [27], CALM2 (calmodulin 2) gene was downregulated.

The regulation of various calcium-associated genes suggested the critical role of calcium signaling in the CS-derived MSC spheroids, which has not yet been reported in any other spheroid systems.

### Regulation of cell adhesion and migration/cytoskeleton-associated genes for MSCs on CS

Genes that were screened out for MSCs on CS and associated with cell adhesion, migration, or cytoskeleton reorganization are displayed in Table 2. MMP1 (matrix metalloproteinase 1) is a kind of interstitial collagenase, and its activity was enhanced in highly migrating MSCs compared with poorly migrating MSCs [28]. MMP3 and MMP10 were also upregulated which have similar substrate specificity. MMP3 is correlated with neuronal migration and neurite outgrowth and is able to activate MMP1 [29]. The migration ability of CS-derived MSC spheroids has been mentioned [5, 9]. The upregulation of these cell adhesion/migration-associated genes agreed with the high cell mobility of MSCs on CS.

**Table 2:** **The group of adhesion and migration/cytoskeleton-associated genes screened from microarrays**

Gene symbol	Gene full name	Ratio (MA)	Ratio (qRT-PCR)
ITGB8	integrin, beta 8	5.48	5.28 ± 2.30*
ITGA2	integrin, alpha 2	2.73	-
ITGA10	integrin, alpha 10	2.66	-
ITGA11	integrin, alpha 11	2.68	-
ITGB1	integrin, beta 1	0.32	-
ITGA6	integrin, alpha 6	0.33	-
MMP10	matrix metallopeptidase 10 (stromelysin 2)	14.14	39.10 ± 12.35*
MMP1	matrix metallopeptidase 1 (interstitial collagenase)	3.71	2.75 ± 0.72*
MMP3	matrix metallopeptidase 3 (stromelysin 1, progelatinase)	2.23	-
CDH18	cadherin 18, type 2	3.14	5.46 ± 3.08*
PCDH18	protocadherin 18	8.20	4.18 ± 1.70*
PECAM1	platelet/endothelial cell adhesion molecule 1	3.25	2.68 ± 1.04*
NOTCH3	notch 3	2.24	2.40 ± 0.43*
DLL1	delta-like 1 (Drosophila)	2.51	2.27 ± 0.19*
EPHA7	EPH receptor A7	20.49	12.34 ± 4.95*
SGCG	sarcoglycan, gamma (35 kDa dystrophin-associated glycoprotein)	2.05	-
SHC4	SHC (Src homology 2 domain containing) family, member 4	2.36	-
PTPRB	protein tyrosine phosphatase, receptor type, B	2.55	-
SORBS2	sorbin and SH3 domain containing 2	8.31	7.11 ± 1.90*
DMD	dystrophin	0.48	0.38 ± 0.06*
CCBE1	collagen and calcium binding EGF domains 1	0.25	0.19 ± 0.07*
HMMR	hyaluronan-mediated motility receptor (RHAMM)	0.23	0.29 ± 0.06*
CMKLR1	chemokine-like receptor 1	49.04	93.69 ± 36.86*
CXCR4	chemokine (C-X-C motif) receptor 4	13.40	16.61 ± 4.90*
CXCR7	chemokine (C-X-C motif) receptor 7	4.60	10.78 ± 4.99*
CXCL10	chemokine (C-X-C motif) ligand 10	4.10	8.99 ± 4.86*
CCL2	chemokine (C-C motif) ligand 2	3.44	-
CCL7	chemokine (C-C motif) ligand 7	2.83	-

*P < 0.05 between CS and TCPS groups (n = 5).

On the other hand, a variety of genes that controls cell-to-cell adhesion were upregulated for MSCs on CS, including cadherins, cell adhesion molecules (CAMs), Notch, and ephrin receptor. CDH18 (cadherin 18, also named as cadherin 14) is a Ca^2+^-dependent cell-cell adhesion molecule, and expresses in the central nervous system [30]. PCDH18 (protocadherin 18) is also a member of cadherin family, and has a role in embryo development [31]. PECAM1 (platelet endothelial cell adhesion molecule, also known as CD31) is an endothelial cell marker [32]. The expression of NOTCH3 (Notch receptor 3) and its ligand, DLL1 (delta-like protein 1), were both enhanced for MSC growth on CS. Notch signaling pathway is critical for cell fate decisions including proliferation, lineage commitment, and terminal differentiation in many adult stem cell types [33]. EphA7 (Ephrin type-A receptor 7) can bind to cell surface-associated ephrin ligands on neighboring cells to generate bidirectional signals that affect both the receptor-expressing and ephrin-expressing cells [34]. Based on the literature, EphA7 is an axon guidance receptor important for the development of cortical circuits [35]. The enhancement in these cell-cell adhesion genes may provide better cell-cell communication and coordination during spheroid formation.

The upregulation of chemokines and their receptors for MSCs grown on CS vs. TCPS is of particular interest. Among them, the gene encoding CMKLR1 (chemokine-like receptor 1, also as chemerin Receptor 23, ChemR23) was upregulated superbly (~50 times). CMKLR1 was reported as a multifunctional receptor which can bind with the proinflammatory chemokine, chemerin, or with the anti-inflammatory lipid mediator, resolving E1 (RvE1, a bioactive oxygenated product of the essential fatty acid, eicosapentaenoic acid) [36]. Chemerin/CMKLR1 interaction was also reported to promote adipogenesis and angiogenesis [36]. Other upregulated chemokine receptors or ligands included the CXCR4 and CXCR7 (CXC motif chemokine receptor 4 and 7), which are the receptors of stromal derived factor-1 (SDF1 or CXCL12). CXCR4 is one of the most studied chemokine receptors that play an important role in cell migration, proliferation, and differentiation [37]. The CXCL10 (CXC motif chemokine ligand 10) is a ligand for another CXC motif chemokine receptor, CXCR3, which was reported to crosstalk with CXCR4 and CXCR7 [37]. The CCL2 and CCL7 [C-C motif chemokine ligand 2 and 7, also referred as monocyte chemotactic protein 1 and 3 (MCP-1 and MCP-3)] are important homing factors for MSCs [38, 39].

### Cell fate decision in MSC spheroids

The gene expression for a group of growth factors and receptors was modulated in MSCs on CS vs. TCPS, as listed in Table 3. These included TGF-β3 (transforming growth factor beta 3), BMP2 (bone morphogenetic protein 2), HGF (hepatocyte growth factor), IGF1R (insulin-like growth factor 1 receptor), KDR (kinase insert domain receptor, also known as vascular endothelial growth factor receptor 2, VEGFR2) and KIT (the stem cell factor receptor, also known as CD117). The regulation of these genes may influence the function and fate of stem cells.

**Table 3:** **The group of development-associated genes screened from microarrays**

Gene symbol	Gene full name	Ratio (MA)	Ratio (qRT-PCR)
TGFB3	transforming growth factor, beta 3	2.83	2.55 ± 0.89*
BMP2	bone morphogenetic protein 2	3.00	9.79 ± 3.33*
HGF	hepatocyte growth factor (hepapoietin A; scatter factor)	2.56	7.89 ± 2.73*
IGF1R	insulin-like growth factor 1 receptor	2.16	2.26 ± 0.71*
INSR	insulin receptor	2.02	-
KDR	kinase insert domain receptor (a type III receptor tyrosine kinase)	2.19	5.30 ± 1.48*
KIT	v-kit Hardy-Zuckerman 4 feline sarcoma viral oncogene homolog	4.50	6.21 ± 1.23*
EGF	epidermal growth factor	0.21	-
HBEGF	heparin-binding EGF-like growth factor	0.35	-
CTGF	connective tissue growth factor	0.16	-
BDNF	brain-derived neurotrophic factor	0.13	0.33 ± 0.10*
GHR	growth hormone receptor	0.44	-
WLS	wntless homolog	3.31	3.95 ± 0.71*
LEF1	lymphoid enhancer-binding factor 1	7.09	2.14 ± 0.16*
TCF7	transcription factor 7 (T-cell specific, HMG-box)	3.79	1.35 ± 0.03*
DAAM1	dishevelled associated activator of morphogenesis 1	3.17	3.61 ± 0.60*
WNT2	wingless-type MMTV integration site family member 2	3.83	4.66 ± 0.85*
LRP4	low density lipoprotein receptor-related protein 4	2.28	-
DGKG	diacylglycerol kinase, gamma 90 kDa	2.56	-
CXXC4	CXXC finger protein 4	14.52	7.21 ± 1.02*
RARB	retinoic acid receptor, beta	4.50	1.68 ± 0.16*
EGR2	early growth response 2	26.55	25.32 ± 4.83*

* P < 0.05 between CS and TCPS groups (n = 5).

Several WNT signaling related genes were also enhanced for MSCs on CS including the WLS (Wntless protein), WNT2 (wingless-type MMTV integration site family member 2), LEF1 (lymphoid enhancer-binding factor 1), TCF7 (transcription factor 7), DAAM1 (dishevelled associated activator of morphogenesis 1), and CXXC4 (CXXC finger protein 4). The WNT proteins and WNT signaling pathway are known to control cell specification and fate during embryonic development and adult tissue homeostasis. WNT2, a member of WNT family, can promote the earliest aspects of lung airway smooth muscle development [40], and accelerate cardiac myocyte differentiation from ES-cell derived mesodermal cells through the non-canonical WNT pathway [41]. WLS is a multipass transmembrane protein, and was found to control the secretion of WNT proteins [42]. The TCF/LEF family is the downstream proteins in the canonical WNT/ β-catenin pathway. In response to WNT signals, TCF/LEF members present as a switch to modulate the transcription of numerous target genes from repression to activation as binding with β-catenin [43]. DAAM1 was identified as a downstream protein interacting with Dishevelled (Dvl), which mediates the non-canonical Wnt/planar cell polarity (PCP) signaling pathway. A study indicated that DAAM1 may play a crucial role in regulating the actin cytoskeleton and tissue morphogenesis [44]. On the other hand, one of the negative regulators of WNT/ β-catenin signaling pathway, CXXC4, was also enhanced for MSCs on CS [45].

Two other development-related genes worth mentioning are RARB (retinoic acid receptor beta) and EGR2 (early growth response 2). RARB is a nuclear receptor for retinoic acid (RA) which is a vitamin A-derived, non-peptidic, small lipophilic molecule. The RA signaling during embryo development has been extensively investigated [46]. EGR2 is a zinc-finger transcription factor of the early growth response gene (EGR) family that has critical functions in hindbrain development and myelination of the peripheral nervous system [47]. The gene expression of EGR2 may be regulated by TGF-β3 [48].

### Antiinflammatory and antitumor properties of MSCs on CS

Genes upregulated for MSCs growth on CS and encoding cytokines or their receptors are listed in Table 4. Among them, a small number of proinflammatory cytokines were upregulated, which included IL1A (interleukin 1 alpha), IL1B (interleukin 1 beta), IL33 (interleukin 33) [49], and TNFSF13B (tumor necrosis factor ligand superfamily member 13B, also as B-cell activating factor, BAFF) [50]. Many antiinflammatory genes were enhanced to even higher expression levels as compared with those of proinflammatory ones. These genes include the IL1RN (interleukin 1 receptor antagonist) [51], IL4I1 (interleukin 4 induced gene 1) [52], LIF (leukemia inhibitory factor) [53] and its common receptor subunit, IL6ST (interleukin 6 signal transducer, also named as glycoprotein 130, gp130) [54], and TNFAIP6 (tumor necrosis factor, alpha-induced protein 6, also as TNFα stimulated gene/protein 6, TSG6) [55]. The TNFSF9 (tumor necrosis factor superfamily member 9) was also upregulated which can mediate both immune suppression and immune stimulation through the CD137 receptor/ligand system [56]. Another upregulated antiinflammatory-associated gene was PTGS2 (prostaglandin-endoperoxide synthase 2, also named as cyclooxygenase-2, COX-2), which is the rate-limiting enzyme for arachidonic acid metabolic transformation into prostanoids during eicosanoid synthesis in response to inflammatory stimuli [57]. Besides, the above-mentioned LIF, TGF-β and HGF, are considered as factors associated with the immunomodulatory property of MSCs [58]. This aspect of MSCs is important in clinical application for graft-versus-host and autoimmune diseases.

**Table 4:** **The group of antiinflammatory and antitumor genes screened from microarrays**

Gene symbol	Gene full name	Ratio (MA)	Ratio (qRT-PCR)
IL1A	interleukin 1, alpha	2.12	3.48 ± 1.09*
IL1B	interleukin 1, beta	2.12	-
IL1RN	interleukin 1 receptor antagonist	6.33	5.72 ± 0.88*
IL33	interleukin 33	3.85	-
IRAK2	interleukin-1 receptor-associated kinase 2	2.50	-
IL4I1	interleukin 4 induced gene 1	2.34	-
IL24	interleukin 24	4.77	18.59 ± 10.05*
IL6ST	interleukin 6 signal transducer (gp130, oncostatin M receptor)	4.07	-
LIF	leukemia inhibitory factor	2.02	3.70 ± 1.77*
TNFAIP8L3	tumor necrosis factor, alpha-induced protein 8-like 3	13.87	10.28 ± 2.41*
TNFAIP8	tumor necrosis factor, alpha-induced protein 8	3.89	7.02 ± 1.75*
TNFAIP6	tumor necrosis factor, alpha-induced protein 6	3.31	-
TNFSF13B	tumor necrosis factor (ligand) superfamily, member 13b	2.50	-
TNFSF9	tumor necrosis factor (ligand) superfamily, member 9	2.36	-
TNFRSF11B	tumor necrosis factor receptor superfamily, member 11b	2.23	-
C1QTNF6	C1q and tumor necrosis factor related protein 6	2.43	-
PTGS2	prostaglandin-endoperoxide synthase 2 (prostaglandin G/H synthase and cyclooxygenase)	4.71	15.09 ± 1.73*
TP53	tumor protein p53	2.11	1.48 ± 0.03*

*P < 0.05 between CS and TCPS groups (n = 5).

A few antitumor genes were upregulated for MSCs on CS. Of interest to note is the high gene expression level of IL24 (interleukin 24) observed for MSCs grown on CS vs. TCPS. IL24 is considered as a tumor suppressor and can selectively induce apoptosis in cancer cells without affecting normal cells [59, 60]. TP53 ( tumor protein p53) is another upregulated tumor suppressor gene that involves in various critical cellular functions such as proliferation, cell cycle arrest, apoptosis, and DNA repair mechanisms [61]. The members of TNFα-induced protein 8 (TNFAIP8) family were upregulated. TNFAIP8 was reported as an apoptosis regulator, while the gene encoding TNFAIP8L3 (TNFα-induced protein 8 like 3) is still unknown for its biological function [62].

### Regulation of aryl hydrocarbon receptor (AHR) pathway

The upregulation of aryl hydrocarbon receptor (AHR) system was also observed and displayed in Table 5. AHR is a ligand-activated transcription factor activated by endogenous physical ligands. AHR is involved in a variety of toxicity mechanisms as well as in endogenous biological functions. ARNT (AHR nuclear translocator) was also upregulated, which can transduce the AHR signaling and promote the expression of target genes (e.g., cytochrome P450s) [63]. Cytochrome P450s (CYPs) have been identified as the functional enzymes that catalyze the metabolic activation and detoxification of a variety of xenobiotics [64]. The expressions of CYP1B1 (cytochrome P450, family 1, subfamily B, polypeptide 1), CYP3A5 (cytochrome P450, family 3, subfamily A, polypeptide 5), and CYP19A1 (cytochrome P450, family 19, subfamily A, polypeptide 1) were upregulated for MSCs on CS. CYPs play a critical role in drug metabolism, and therefore cells with appreciated CYP activities can be used for risk assessment of drug-induced hepatotoxicity [65]. A recent study showed that the expression and activities of CYPs were enhanced by culturing the transfected human dermal fibroblasts as spheroids [65], which was in line with our results.

**Table 5:** **Other significant genes screened from microarrays**

Gene symbol	Gene full name	Ratio (MA)	Ratio (qRT-PCR)
AHR	aryl hydrocarbon receptor	3.48	4.29 ± 1.33*
ARNT2	aryl-hydrocarbon receptor nuclear translocator 2	2.12	-
CYP1B1	cytochrome P450, family 1, subfamily B, polypeptide 1	7.66	10.91 ± 0.57*
CYP19A1	cytochrome P450, family 19, subfamily A, polypeptide 1	5.48	-
CYP3A5	cytochrome P450, family 3, subfamily A, polypeptide 5	3.95	-
FOXO1	forkhead box O1	2.22	1.75 ± 0.17*
HS3ST1	heparan sulfate (glucosamine) 3-O-sulfotransferase 1	16.47	9.06 ± 1.16*
G0S2	G0/G1switch 2	7.67	-
CDKN2B	cyclin-dependent kinase inhibitor 2B (p15, inhibits CDK4)	3.65	6.00 ± 1.73*
INHBB	inhibin, beta B	0.16	0.26 ± 0.13*

* P < 0.05 between CS and TCPS groups (n = 5).

### Substrate-dependent nature of gene upregulation for 3D spheroids

The expressions of special interested genes for MSC spheroids grown on CS and those derived on a non-adherent (polyvinyl alcohol, PVA) substrate were compared by qRT-PCR. The mechanism driving spheroid formation on the non-adherent PVA is similar to that in suspension culture (where a low-attachment dish or flask is used). The self-made PVA substrate, however, is more chemically defined. The results (see Additional file 1: Figure S2) showed significantly higher expression levels of genes including LIF, IL24, TP53, TGF-β3, PDGFRA, and PTGS2 for MSCs grown on CS vs. PVA. The enhanced gene expressions may be attributed to the greater cell-substrate interaction for MSCs on CS through the upregulation of the calcium-associated genes.

Furthermore, the high expression levels for the above-mentioned genes in CS-derived spheroids were maintained or even further enhanced at 72 h as compared with those at 16 h. The sustained regulation of these genes by CS vs. the transient upregulation by PVA suggested the importance of cell-substrate interaction in gene regulation. The differential gene expression in different substrate-derived MSC spheroids also suggested substrate-dependent gene regulation and the critical role of culture substrates in influencing the cell functions and fates even in 3D spheroids.

## Discussion and conclusions

The gene expression profiles of CS substrate-derived 3D MSC spheroids vs. 2D cultured MSCs were cross-analyzed by mRNA as well as miRNA microarrays and confirmed by qRT-PCR measurement. The critical role of calcium signaling in CS substrate-derived MSC spheroids was justified by the upregulation of various calcium-associated genes, which has not yet been reported in any other spheroid systems. The unique role of calcium in self-assembled spheroids may be related to the observation that the surface-bound calcium on CS may be translocated into MSCs [9].

Several kinds of integrin subunit which participate in the processing of cell adhesion or migration were modulated. Besides, members of matrix metalloproteinases (MMPs) were upregulated for MSCs grown on CS. MMPs are proteolytic enzymes that degrade various components of the extracellular matrix (ECM). The proteolytic effects of MMPs play an important role in vascular remodeling, cellular migration, and the processing of ECM proteins and adhesion molecules [66]. The modified chemotactic function of the migrating MSC spheroids was also verified by the upregulation of many chemokines and their receptors. Since the chemotactic function is critical for the therapeutic performance of MSCs, the migration and chemotaxis of MSC spheroids and their link to homing phenomena deserve further investigations.

The multilineage differentiation capacities as well as antiinflammatory and antitumor properties of MSCs may be enhanced after forming spheroids on CS. The antiinflammatory and antitumor properties have been reported in MSC spheroids generated by hanging drop [2], including the upregulated gene expression of TNFAIP6 and IL24, which were also observed in CS-derived MSC spheroids. The favorable chondrogenic and osteogenic differentiation capacities previously demonstrated for CS-derived MSC spheroids [5, 7] may be associated with the induced expression of TGF-β3 and BMP2 genes. On the other hand, HGF, EGR2, MMP3, and EPHA7 are involved in the development of nervous system [29, 47, 67, 68]. The upregulation of these genes may suggest the enhanced transdifferentiation ability of CS-derived MSC spheroids. Most of all, the upregulation of WNT related genes suggested a profound influence of CS on the fate decision of MSCs. The regulatory changes in the expression of these genes were significantly greater for MSC spheroids derived on CS substrates than those derived on the non-adherent PVA substrates. The critical importance of substrates in stem cell culture, even in the circumstance of 3D spheroid culture, was substantiated in this study. The distinct gene expression profiles on different substrates were in line with the significant substrate-dependent alterations in cell-cell interaction (e.g. cadherins, CAMs, and Notch, etc) and cell-substrate interaction (e.g. integrins) based on the gene analysis.

Finally, it has been mentioned that cells in the core of a 3D spheroid may be exposed to mild hypoxia [69]. The hypoxic environment may resemble the natural niche of MSCs (e.g. O_2_ tension ~1−7% in bone morrow) more than the normal culture condition (21% O_2_) [70]. A recent literature has demonstrated that MSCs can benefit from hypoxia to inhibit the senescence, increase the proliferation, and enhance the differentiation potential along the mesenchymal lineages [70]. The hypoxia-inducible factor 1α(HIF-1α) signaling pathway was proposed to be involved in the modulation mechanism of hypoxia effect [70]. Spheroids generated by suspension culture could precondition the human adipose-derived stromal cells [69] and umbilical vein endothelial cells [71] to hypoxia environment, leading to upregulations of HIF-1α and angiogenesis. Another recent literature showed that forming 3D spheroids of human gingiva-derived MSCs by suspension culture upregulated many hypoxia-responsive genes, such as HIF-1α, VEGF, SDF-1α, and CXCR4 [72]. The apoptosis signal-regulating kinase 1 (ASK1) and its downstream proteins, the p38α mitogen-activated protein kinase (MAPK) family, act as sensors of oxidative stress [73, 74]. In our study, the upregulation of CXCR4 was observed for MSC spheroids grown on CS. On the other hand, neither the oxidative stress-sensitive genes (including HIF-1α, ASK1, and p38α MAPK) nor the oxidative stress-associated microRNA (such as miR-200a [74], miR-125b [75] miR-30b [76], miR-144 [77] and miR-27b [78]) were screened out by the mRNA and miRNA microarrays. These results suggested that the oxidative stress and the associated genes may not be activated during the formation of MSC spheroids on CS, and the modulating mechanism of the development-associated genes such as CXCR4 in CS-derived spheroids may be distinct from that in spheroids derived on a non-adherent substrate. This finding reinforces the uniqueness of culture substrates as a microenvironment to predefine the properties of 3D stem cell spheroids.

Although MSCs also formed spheroids on non-adherent substrate (such as PVA), the forming process and the gene regulation profile were not the same as those on CS. On the other hand, MSCs showed similar features of spheroid formation and calcium-related cell behavior on CS substrates even when they were isolated from different tissue sources (adipose, placenta, or umbilical cord) or species (human or rat) [5, 9]. The capacity of MSCs to form spheroids on CS was more influenced by their stemness [5], and the surface-bound calcium on the substrate [9]. In this study, we observed that a few genes were regulated during spheroid formation which may participate in calcium signaling pathway. However, the critical genes that turn on the mechanism as well as the link between calcium regulation and the genes involved in different cell functions (adhesion, migration, antiinflammatory, and differentiation) remains unknown. These issues are interesting and worthy of further investigations. The gene regulation profile screened by the cross-correlation analysis described here may provide helpful information for studying these unique substrate-induced MSC spheroids.

## Methods

### Isolation and culture of MSCs

All human subjects and protocols involved were approved by the institutional review board of Chang Gung Memorial Hospital (IRB#92-176). The fresh umbilical cords were collected at the hospital after obtaining written informed consent from each donor participating in this study. The informed consent is always obtained from the mother. The blood vessels were removed by washing with PBS. The cleaned-up tissue was sliced into small pieces and digested with 0.05% trypsin and 300 U/ml collagenase in alpha minimum essential medium (α-MEM, Gibco) for 1 h at 37°C. Cells were gathered from pellets after centrifugation and incubated at 37°C with 5% CO_2_. The culture medium consisted of α-MEM supplemented with 10% fetal bovine serum (Hyclone), 10 mg/l penicillin-streptomycin, and 10 mg/l l-glutamine (Gibco). On the next day, non-adherent cells were removed. The medium was refreshed two times every week. Cells of the 2nd to the 6th passages were used in this study.

### Analysis of surface markers for the human MSCs

Surface markers for human umbilical cord MSCs were quantified by flow cytometry using CD13, CD14, CD29, CD31, CD34, CD44, CD45, CD56, CD59, CD61, CD71, CD105, CD106, CD133, HLA-ABC, HLA-DR (all from BioLegend), CD73 (BD Pharmingen), and CD90 (Serotec) antibodies. MSCs (5×10^5^ cells) were washed twice with PBS, resuspended in 100 μl of PBS containing monoclonal antibodies, and incubated for 30 min at 4°C. These cells were then washed twice and resuspended in 500 μl of PBS. Fluorescence analysis was performed with a flow cytometer (FACS Caliber, BD). The non-specific binding of the fluorescein isothiocyanate (FITC) and phosphatidyl ethanolamine (PE) conjugates were determined in control samples using a mouse IgG1-FITC and IgG1-PE negative control (Serotec). Analysis was conducted using the WinMDI 2.9 software.

### Preparation of chitosan (CS) membranes as the culture substrate

CS powder (molecular weight ~416 kDa, 77.7% deacetylation, Fluka) was dissolved and stirred in 1% aqueous acetic acid solution for 24 h at room temperature to obtain a 1% CS solution. The 1% CS solution was casted on 6-well tissue culture plate (1.5 ml/ well) or 15 mm microscope coverslip glass (300 μl/ slip) and air-dried for 2 days. The CS substrates were treated with 0.5 N NaOH in 75% ethanol for 5 minutes, and then washed extensively by distilled water. These CS substrates were further antiseptically rinsed with 75% ethanol and washed by phosphate buffer saline (PBS) before use. The static water contact angle of CS membranes were determined by a contact angle meter (FTA, USA). The surface zeta potential was determined by electrophoretic light scattering using the Delsa Nano C Analyzer (Beckman Coulter, USA) with a flat solid cell. To analysis the amount of surface-bound calcium on substrates, CS-coated coverslip glass was placed into the well of a 24-well tissue culture plate where 1 ml of culture medium was added. After incubation at 37°C for 24 h or 72 h, the medium was collected for later analysis of the free calcium ion remained in the bulk solution. A blank well (TCPS) was used as the control. The concentration of calcium in each of the collected solution was measured by the atomic absorption (AA) spectrometry (iCE 3300; Thermo Scientific, USA). The content of surface-bound calcium was calculated by subtracting the amount of calcium remained in the collected solution from those in stock culture medium.

### MSC culture on CS membranes

MSCs (2.5 × 10^5^ cells per well for 6-well plate) were seeded in each well and the morphology of cells on the membranes was observed by an inverted microscope (Leica DMIRB). Cells seeded in the culture well (tissue culture polystyrene, TCPS) served as the control. The average diameters of spheroids were quantified from the images. The cell viability in MSC spheroids was determined by using propidium iodide (PI) (Sigma) staining and flow cytometry. After grown on CS for 24 h, MSC spheroids were collected and dissociated in 0.25% trypsin-EDTA solution for 10 min at room temperature. These cells were then washed and resuspended in 500 μl of PBS. The solution of PI (concentration 2 mg/ml) was added to cell suspension before the analysis by the flow cytometer. The percentage of cells without being stained by PI was defined as the cell viability.

### Analysis of gene and miRNA expression microarray

To understand the signaling events involved in the spheroid formation on CS, MSCs of the 6th passage were cultured on TCPS or CS substrates for 16 h. Total RNA of these MSCs were extracted, and then analyzed by gene and miRNA expression microarrays. As for the analysis of gene expression, treatment RNA (CS substrates) was labeled by Cy5 and RNA from human reference RNA (TCPS) was labeled by Cy3. Cy-labeled cRNA (2 μg) was fragmented to an average size of about 50-100 nucleotides by incubation with fragmentation buffer at 60°C for 30 min. Correspondingly, fragmented-labeled cRNA was then pooled and hybridized to the Human 1A (version 2) gene expression microarray (Agilent Technologies) at 60°C for 17 h. After washing and drying in nitrogen, the microarrays were scanned with the Agilent microarray scanner at 535 nm for Cy3 and at 625 nm for Cy5. Scanned images were analyzed using Feature Extraction software 10.7 (Agilent Technologies). Only the microarray images with signal significant ratios > 3 in either the Cy3 or Cy5 channel were retrieved for further analysis.

On the other hand, the miRNA was isolated by using miRNeasy Mini kits (Qiagen) followed by quality checks of both total RNA and small RNA using a 2100 Bioanalyzer and software which detected 28S and 18S ribosomal RNA ratios, generated a RNA Integrity Number (RIN), concentration of sample, and ribosomal ratio. Only samples with 28S/18S > 1.2, RIN > 8, and detectable miRNA were used for this study. The Agilent customer Human R16 miRNA array was employed for this study following manufacturer's protocols. The screened data of miRNA microarray were analyzed by the software, GeneSpring 7.3.1 (Agilent Technologies). The miRNA microarray images with signal ratio greater or lower than three times were screened out and defined as the normalized miRNA profile for further analysis.

### qRT-PCR confirmation for the genes screened by cross-correlation analysis of microarrays

In order to further validate the results derived from microarrays, qRT-PCR was performed for special interested genes. In brief, total RNA of cells at the end of the culture period was extracted by the Trizol® reagent (Invitrogen) according to the manufacturer’s instructions. Human MSCs cDNA synthesis and amplification via qRT-PCR were performed using the RevertAidTM First Strand cDNA Synthesis Kit (Thermo, Fermentas). Paired forward and reverse primers were designed from UniSTS database in National Center for Biotechnology Information. The 100 ng of cDNA was used for quantitative real-time PCR using the GM SYBR qPCR Kits (GeneMark, Taiwan) with 150 nM targeted gene oligonucleotide primer pairs. 40 cycles of PCR consisting of denaturing at 95°C for 2 s (3 min in the first cycle), annealing and extension for 30 s were performed by a Chrom4 Thermal Cycler System (MJ Research). The value of each sample was normalized to the expression of the GAPDH housekeeping gene in the same sample. The primer sequences for each gene used in this study are shown in Additional file 4: Table S1).

### Statistical analysis

Multiple samples were used in each experiment. Numerical values were expressed as the mean ± standard deviation. Statistical differences among the experimental groups were evaluated by two-tailed student’s *t*-test. A significant difference was considered when P ≤ 0.05. In all studies, three independent experiments were performed for each type of experiments.

## Electronic supplementary material

Additional file 1: Figure S1: Flow cytometric analysis of various surface markers for human umbilical cord MSCs. **Figure S2.** The relative ratio of gene expressions for MSCs on CS or PVA (non-adherent) vs. TCPS after 72 h of culture. (DOC 796 KB)

Additional file 2: **Cross-correlation analysis.** The list of genes screened by the cross-correlation analysis as well as their expression ratios. (XLS 5 MB)

Additional file 3: **Gene enrichment analysis.** Results from gene enrichment analysis in terms of gene ontology (GO terms) and Kyoto Encyclopedia of Genes and Genomes (KEGG) pathway maps, using the DAVID program. (XLS 236 KB)

Additional file 4: Table S1: Information for each primer used for the real-time RT-PCR. (DOC 76 KB)
