# Supplementary material for: Substrate-dependent gene regulation of self-assembled human MSC spheroids on chitosan membranes
Source: BMC Genomics. 2014 Jan 5;15(1):10. doi: 10.1186/1471-2164-15-10 (PMC4046657; doi:10.1186/1471-2164-15-10)
Supplement: Supplementary file 1 — Additional file 1: Figure S1: Flow cytometric analysis of various surface markers for human umbilical cord MSCs. Figure S2. The relative ratio of gene expressions for MSCs on CS or PVA (non-adherent) vs. TCPS after 72 h of culture. (DOC 796 KB) [file 12864_2013_5655_MOESM1_ESM.doc]

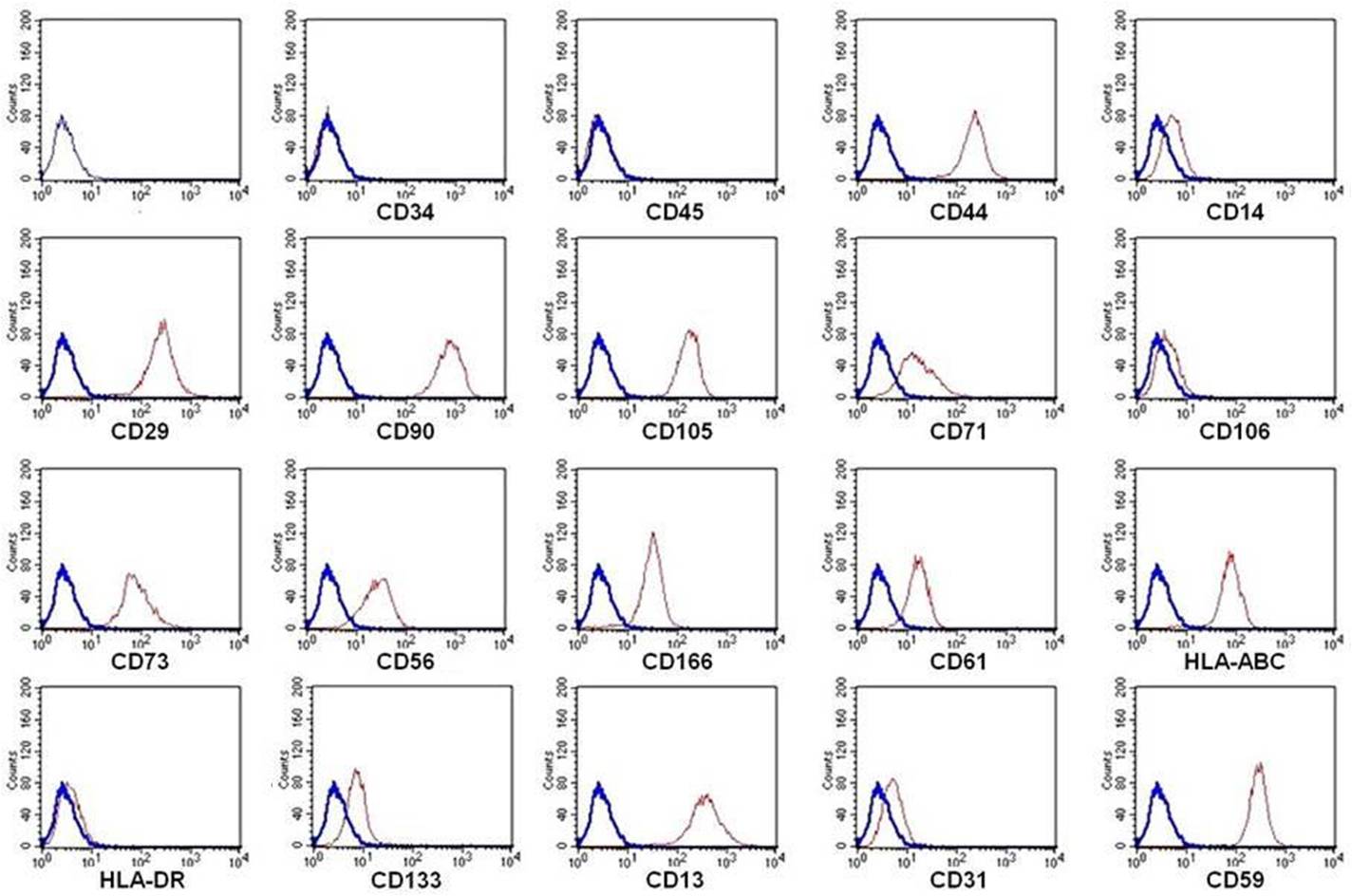


**Figure S1.** Flow cytometric analysis of various surface markers for human umbilical cord MSCs. The results showed that cells were negative for CD14, CD34, CD45, CD106, CD133, and HLA-DR, and were positive for CD13, CD29, CD44, CD56, CD59, CD61, CD71, CD73, CD90, CD105, CD166, and HLA-ABC.

**Figure S2.** The relative ratio of gene expressions for MSCs on CS or PVA (non-adherent) vs. TCPS after 72 h of culture. * P<0.05 among the indicated groups (n=4).
