# Supplementary material for: Substrate-dependent gene regulation of self-assembled human MSC spheroids on chitosan membranes
Source: BMC Genomics. 2014 Jan 5;15(1):10. doi: 10.1186/1471-2164-15-10 (PMC4046657; doi:10.1186/1471-2164-15-10)
Supplement: Supplementary file 4 — Additional file 4: Table S1: Information for each primer used for the real-time RT-PCR. (DOC 76 KB) [file 12864_2013_5655_MOESM4_ESM.doc]

**Table S1. Information for each primer used for the quantitative RT-PCR.**

| Gene | Primer sequences | Primer annealing  temperature (°C) |
| --- | --- | --- |
| GAPDH | Forward: GAAGGTGAAGGTCGGAGTC  Reverse: GAAGATGGTGATGGGATTTC | 62 |
| ATP2B1 | Forward: ACTGGGCCTAAAACCATGA Reverse: CTGACCCAGAAAGGCAGTT | 58 |
| ATP2B4 | Forward: CCTATCTGCGTGCTAATTTG Reverse: AGGAGAGATGGAAGGAGATG | 58 |
| SLC8A1 | Forward: GTGGAACATGGAAAGTAAAGGA Reverse: CCCACCTAACGTGGCCAT | 62 |
| TRPA1 | Forward: CCAGGTTAGAATCGAAAACAGG Reverse: AATTTTCAGTCCAGTAAGGGAGC | 60 |
| TRPC4 | Forward: ACGTAATCTTCGTGGGTGACTGT Reverse: CTGCAGGACCACTTGAGAGAAAT | 60 |
| HTR2A | Forward: GTGGCAACTGTGGAAGGC Reverse: GAAAGCTCACAGCTGAAATGC | 60 |
| PDGFRA | Forward: AAATGGGTGCTAAATTGATTGG Reverse: GCACATCTTTAGCAGGAGCC | 62 |
| GPR68 | Forward: CTACATAAATCCATTACAGAGTC Reverse: GATTTTACTGGGTCAGTG | 62 |
| F2R | Forward: TCCAAAGAGATACCAAGCA Reverse: TCAGTGAAGATTTACTGTCATTGTT | 60 |
| MAP3K8 | Forward: GAATTAGAAGCCATCTGACAGCA Reverse: GAAAGATTCATGAAGGCATTTGA | 60 |
| ITPR1 | Forward: TTCATTCCTTTTTTGAAAGAATAAA Reverse: AATTAAACCACTGGCTCAAACA | 60 |
| PLA2G4A | Forward: TGGCCACTGAAGATTAATTGC  Reverse: TCCCGACTCTTGTGCACTC | 62 |
| RASGRP3 | Forward: TTAACCCAGTCAGAAGGGACA  Reverse: TCCTTCCCACTACACAGTTGC | 58 |
| CALM2 | Forward: CAGCCAAGCGCAAAGCAGGCG  Reverse: CAAGAGATCAAGGAAAGTGGGCG | 62 |
| ITGB8 | Forward: CAGTAATCCCTGCACTGGAC  Reverse: AAGGTTGTATCTGTTCACCTCTTG | 60 |
| MMP10 | Forward: CTAGGCGAGATAGGGGG  Reverse: TTAATTCTGTTCAGTGCAATTC | 60 |
| MMP1 | Forward: GTGTGTCTCCTTCGCACACATCTTG  Reverse: GAGTCCTTGCCCTTCCAGAAAGCC | 62 |
| CDH18 | Forward: CCATCATATCCAGCCAAGGT  Reverse: ATTCACCTGATGAGAATTGCTG | 58 |
| PCDH18 | Forward: AATGCAACTATTTGGCAATGC  Reverse: CAGTGAACTGGTGGCAGAGA | 62 |
| PECAM1 | Forward: TAATACAACATCCACGAGGGTCC  Reverse: ACAAAATTGCTTGCTAAAGAAGTGG | 62 |
| NOTCH3 | Forward: TGAAGACAGCTCCCACTACT  Reverse: ACGTCGTCCTCACAGTTATC | 62 |
| DLL1 | Forward: AGTCGGTGTACGTCATATCC  Reverse: GTCTGAACTCGGTTTCTCAG | 62 |
| EPHA7 | Forward: ACCATCAATGTATCACCTTCTAAAA  Reverse: AGGCATGTAAAATTTTATTTATGGG | 62 |
| SORBS2 | Forward: ACTTTGGTATGTTGCCCAGG  Reverse: CGAATGCTCATTGCCAATC | 62 |
| DMD | Forward: CCCACCTAATCATATTTCCAC  Reverse: GCTAAAAGCCTGAGCAATAC | 62 |
| CCBE1 | Forward: GAGAGTGAAAATCTCCCGC  Reverse: TTTGCCTGTGGATTCAGAAG | 60 |
| HMMR | Forward: AACTTATACAACACTAGTGGGAGTG  Reverse: TAAATTGTGTTGTCTGAGGCTATGG | 62 |
| CMKLR1 | Forward: TGATCCTCACTGTGGAACCC  Reverse: CTTTGAGTCAGTCAAGGCTGG | 62 |
| CXCR4 | Forward: ACGTAAAGCTAGAAATGATCCCC  Reverse: GTACACTGTAGGTGCTGAAATCAAC | 60 |
| CXCR7 | Forward: GTGCAGATTTGCAGTCCAGA  Reverse: ATCTGTGTGGTGTTTTGTACCG | 60 |
| CXCL10 | Forward: GCTGCTACTACTCCTGTAGGAAGG  Reverse: TGGAAGATGGGAAAGGTGAG | 62 |
| TGFB3 | Forward: GAACTGAGTCAGGGTGCC  Reverse: CGACCAGACCACTTGTTAAA | 62 |
| BMP2 | Forward: AACACGATGTGCATAATTTTGC  Reverse: AAAATTGATATCTCGTGGCCC | 62 |
| HGF | Forward: TGTTCCCTTTTTTGGGTAAGC  Reverse: CCCATTTGCCACAGAAAGTT | 62 |
| IGF1R | Forward: GCTGTCTACCAAGGTGACTTT  Reverse: TGTCTTCAAGTGGGGGTTT | 62 |
| KDR | Forward: GCATCTGAAGGCTCAAACC  Reverse: GAGAATCTGGGCTGTGCTAC | 62 |
| KIT | Forward: TTCTTTCAACTTGCATCCAACTCC  Reverse: TACCTCCCTCTCTTTTTCCAAATC | 60 |
| BDNF | Forward: GGTCGACAGACACTTCATAC  Reverse: CTGGAAATTGGTGGAGGTC | 60 |
| WLS | Forward: TGACTGTGACAACTGCAAATG  Reverse: CAATGGAATGCAACTCCC | 60 |
| LEF1 | Forward: CATACATATTGTCACATTGCTTTCC  Reverse: AATAGAATACGTGATAGATGCTGGG | 62 |
| TCF7 | Forward: CTGGAGAAGCTCAAAGGCC  Reverse: TTGGGTGGTAAGTCAGTGTCC | 60 |
| DAAM1 | Forward: TCCCTTCAGAGAATTCTTCC  Reverse: TGCCAAGTGCTCTTGATAAT | 60 |
| WNT2 | Forward: AGGAACTCTCATTCCCTAAAG  Reverse: GTAACAAGGTGGGGACG | 62 |
| CXXC4 | Forward: CAGATACTTATCAGCTTAAAGAC  Reverse: AGGAGTATATAAAAATGTCTCCT | 58 |
| RARB | Forward: GCCCAAAGAGCACAAACATT  Reverse: CAGACAGTACGTGACATTTCAATG | 62 |
| EGR2 | Forward: GAGACTCAGGCTGATACA  Reverse: TTGGGTTGATAGTCAACT | 58 |
| IL1A | Forward: CATAGCCAGGAAACTCTGC  Reverse: TTGAATGAAACAAGAATGCC | 58 |
| IL1RN | Forward: TACCTGCCAAGAGCGAGG  Reverse: GGGGGTTCTTTCTTCCTCTG | 62 |
| IL24 | Forward: GTGGATTAAAGTGCCCAGC  Reverse: GTCACCATCAGCAAGGTCAG | 62 |
| LIF | Forward: GAGCTGTACCGCATAGTCGT  Reverse: GCGATGATCTGCTTATACTTCC | 62 |
| TNFAIP8L3 | Forward: ATTATTAAGCGAGAAGCTGTCGG  Reverse: CAATTGTTTGTACCCTGGCAGTT | 62 |
| TNFAIP8 | Forward: TCACATAAAATTCACCACTGTAAGCA  Reverse: TTCTGAGAAGCCAGAAATGTGAA | 62 |
| PTGS2 | Forward: AGCCTCGGCCAGATGGC  Reverse: AGGGACAGCCCTTCACG | 58 |
| TP53 | Forward: TTGCCGTCCCAAGCAATGGATGA  Reverse: TCTGGGAAGGGACAGAAGATGAC | 60 |
| AHR | Forward: GTCTATTTATCTCTATCCTG  Reverse: TTTTACTATCTTGAAAGAGCCC | 60 |
| CYP1B1 | Forward: TGTTTCCCCAGAATGTACTTTG  Reverse: TTGTGTGGTTTTAGCTGTGACA | 58 |
| FOXO1 | Forward: GTTCCTGCTGTCAGACAATCTG  Reverse: AAGCTCTAGCTTTTTGCCCC | 60 |
| HS3ST1 | Forward: CACCAGCTCACAGTGGAGAA  Reverse: CTTGTGCTTCTGCATGTGGT | 62 |
| CDKN2B | Forward: CTTCTTGGAATAAATGTCAGG  Reverse: TTTGAAGGATACATGCAAAAC | 60 |
| INHBB | Forward: TGAAACAGAATCTATGGGGAGC  Reverse: CTGCCAACGTTTCAGGGTAT | 62 |
